# Genomic characterization of Vibrio parahaemolyticus strain (AG1) causing translucent post-larvae disease in Penaeus vannamei

**DOI:** 10.1099/mgen.0.001570

**Published:** 2025-12-01

**Authors:** Hung N. Mai, Nguyen Dinh-Hung, Carlos Pantoja Morales, Maiya Matthews, Harris Wright, Arun K. Dhar

**Affiliations:** 1 Aquaculture Pathology Laboratory, School of Animal & Comparative Biomedical Sciences, The University of Arizona, 1117 E Lowell St, Tucson, AZ 85721, USA; 2 Shrimp Improvement Systems, 88081 Old Hwy, Islamorada, FL 33036, USA

**Keywords:** translucent post-larvae disease, *Vibrio*, *Vp*_TPD_

## Abstract

Translucent post-larvae disease (TPD) is a new emerging disease causing massive mortality in shrimp at the larval stage. A highly virulent strain of *Vibrio parahaemolyticus* is reported to be associated with TPD (*Vp*_TPD_). Although few genomes from *Vp*TPD strains isolated from China have been characterized, no comprehensive genomic studies have yet to be carried out for *Vp*TPD strains isolated from samples originating outside of China. This study characterized the whole-genome sequence of *V. parahaemolyticus* strain causing TPD (*Vp*_TPD_). The whole-genome sequence of VpTPD was ~5.5 Mb, consisting of two chromosomes and three plasmids. One of the three plasmids encodes three (VHVP) proteins, causing TPD in shrimp. Genomic characterization revealed that Vibrio High Virulent Protein(VHVP) is a Tc-like toxin complex. Laboratory bioassays conducted using *Vp*_TPD_ revealed that bacterial isolate used in this study causes mortality at the late stage of post-larvae (PL10–PL20). The findings broaden our understanding of the pathogenicity of the *Vp*_TPD_ strain and diagnosis of TPD.

**Impact Statement**

The shrimp industry faces a new emerging disease that causes massive mortality in shrimp at the post-larvae stage, displaying transparent abdominal segments that are referred to as glass-like bodies [6]. The disease is known as translucent post-larvae disease (TPD) [6]. A *Vibrio parahaemolyticus* strain harbouring genes that encode highly virulent proteins is believed to be the causative agent of TPD. However, there is little genomic information available on this *V. parahaemolyticus* strain. This study provides a characterization of the genome of *V. parahaemolyticus*, which causes TPD. The findings of this study contribute to the diagnosis of TPD using histopathology and molecular assays and also enable identifying targets for prophylactic and therapeutic development for TPD.

## Data Summary

The raw reads generated by Illumina and Oxford Nanopore are available in the Sequence Read Archive (PRJNA1193633). The assembled sequences were deposited on GenBank with the accession number shown in Table 1. For analysis, the study used Geneious Prime v2023, Galaxy server (https://usegalaxy.org/), Rapid annotation using subsystem technology (https://rast.nmpdr.org/), https://github.com/kensung-lab/hypo, https://pubmlst.org/multilocus-sequence-typing, https://tygs.dsmz.de/, http://genepi.food.dtu.dk/resfinder, https://github.com/tseemann/abricate and https://cran.r-project.org/web/packages/pheatmap/index.html. The primers and probes were designed using PrimerExpress v3.0. All histology and PCR data are provided in the main text and supplementary files.

**Table 1.** The assembled sequences of AG1 strain

Sequence	GenBank access no.	Length (bp)	Topology	Mean of coverage (X)	
				Short reads	Long reads
Chromosome 1	CP176357	3,493,458	Circular	827.2	176.3
Chromosome 2	CP176358	1,870,850	Circular	792.3	158.5
Plasmid-1 (TPD)	CP176359	69,744	Circular	1694.3	318
Plasmid-2	CP176360	60,722	Circular	1582.6	287.8
Plasmid-3	CP176361	60,518	Circular	1,492	262.6

## Introduction

The shrimp industry is a profitable aquafarming endeavour, bringing billions of dollars to shrimp-producing countries annually [1]. However, diseases caused by microbial pathogens continue to pose threats to the growth and sustainability of the shrimp industry globally. Since the first disease in shrimp was reported at the dawn of the shrimp industry in the early 1970s, shrimp farming has lost billions of dollars annually worldwide [2]. Several microbial pathogens, including viruses, bacteria and fungi, cause shrimp diseases, and the list of microbial pathogens that cause shrimp diseases continues to expand as new diseases emerge [3]. Translucent post-larvae disease (TPD), caused by *Vibrio parahaemolyticus* producing lethal toxins, is a newly emerged disease that is threatening shrimp hatcheries. Several bacterial species of the genus *Vibrio* have caused severe diseases in shrimp farming [4]. While vibriosis compromises general health in hatchery and grow-out ponds, resulting in significant losses in shrimp aquaculture [4], certain species of *Vibrio* have caused large-scale outbreaks in recent years. For example, in 2013 a strain of *V. parahaemolyticus* producing a binary toxin was identified as the aetiologic agent of acute hepatopancreatic necrosis (AHPND) [5]. Since then, several *Vibrio* species have been reported to cause AHPND in shrimp [5]. In 2020, shrimp hatcheries in China experienced an unknown disease that caused large-scale mortalities at the post-larvae stage with translucent or glass bodies as clinical signs. Since then, several studies have been conducted attempting to identify the causative agent of TPD [6,8]. It is now widely accepted that TPD is caused by *V. parahaemolyticus* carrying a plasmid encoding for virulent proteins with a molecular weight >100 kDa [9,10]. Genomic studies reveal that TPD virulent proteins are encoded by three candidate genes (i.e. *vhvp-1*, *-2* and *-3*) located in a ~187 kb plasmid [9]. The VHVP-1 protein comprises 2,544 amino acids containing Tc toxin domains [i.e. TcA receptor-binding domain (TcA_RBD) and a TcA C-terminal TcB-binding domain (TcA_TcB_BD)] and a neuraminidase-like domain. The VHVP-2 is the major virulence factor that causes mortality in shrimp and comprises 1,421 amino acids containing domains similar to a *Salmonella* virulent protein (SpvB) and an insecticidal toxin TcdB middle/N-terminal region [9]. Although it has been reported that VHVP-2 is the virulence factor which causes mortality in shrimp, the results from molecular epidemiology studies reveal that VHVP-1 and -3 likely play roles in enhancing lethality caused by VHVP-2 in shrimp [11]. At the time of drafting this manuscript, only the sequence from JS20200428004-2 originating from China was available in the GenBank (NCBI accession number SRR23329176) under the draft version. Recently, the genome sequence of a few more *Vp*_TPD_ strains (i.e. vp-HL-201910, vp-HL-202005, vp-HL-202006, vp-HL-202008 and vp-HL-202212) has been reported [12]. However, the genome sequence of *Vp*_TPD_ strains isolated outside of China has not been reported. We present here the genome sequence of *Vp_TPD_* originating from Southeast Asia and performed a comparative genome analysis to better understand the pathogenicity of *Vibrio* causing TPD. In addition, we conducted a bioassay to demonstrate the pathogenicity of the isolate and determined the histopathological changes associated with *Vp_TPD_* infection in *Penaeus vannamei* shrimp.

## Methods

### Bacteria

Dead post-larvae were collected from a hatchery experiencing mortality in Southeast Asia, and *V. parahaemolyticus* was isolated on Thiosulfate Citrate Bile Salts Sucrose(TCBS) plates. The colonies were screened for *pirAB*, *vhvp-1* and *-2* using primers and PCR conditions (Table S1, available in the online Supplementary Material) following a published paper [9,13]. The *vhvp-1* and *-2* positive isolates were stored at −80 °C for further studies. The bacterial strain (AG1) used in this study has been reported in our previous study [14].

### Whole-genome sequencing

A *Vp*_TPD_ isolate, AG1, was grown in Tryptic soy broth (TSB) containing 2% NaCl overnight. The bacteria were harvested when OD_600_ reached 3.0. Bacterial cells were pelleted and sent to SeqCenter, LLC (USA) for whole-genome sequencing. Briefly, DNA was extracted from bacteria using ZymoBIOMIC^™^ DNA Miniprep Kit (USA) following the manufacturer’s instructions. For Illumina sequencing, the libraries were prepared using tagmentation-based and PCR-based Illumina DNA Prep kits. Illumina sequencing was performed on an Illumina NovaSeq X Plus sequencer in one or more multiplexed shared-flow-cell runs, producing 2×151 bp paired-end reads. The DNA extracted from AG1 was also subjected to long-read sequencing using Oxford Nanopore Technologies (ONT). The libraries were prepared using the PCR-free ONT Ligation Sequencing Kit (SQK-NBD114.24) with the NEBNext^®^ Companion Module (E7180L) to the manufacturer’s specifications. No additional DNA fragmentation or size selection was performed. Nanopore sequencing was performed on an Oxford Nanopore MinION Mk1B sequencer or a GridION sequencer using R10.4.1 flow cells in one or more multiplexed shared-flow-cell runs. The run design utilized the 400 bp sequencing mode with a minimum read length of 200 bp. Adaptive sampling was not enabled. Guppy1 (v6.5.7) was used for super-accurate base calling, demultiplexing and adapter removal (dna_r10.4.1_e8.2_400bps_modbases_5mc_cg_sup.cfg). The raw data were submitted to the Sequence Read Archive database (PRJNA1193633).

### Genome assembly

The short reads generated from Illumina were trimmed to remove adapters, and low-quality reads were eliminated using Trimmomatic v0.39 via the Galaxy server (https://usegalaxy.org/) [15]. The trimmed reads were assembled *de novo* using SPAdes v3.15.5 (--isolate) via the Galaxy server (https://usegalaxy.org/) [15,16]. The contigs that were longer than 10 kb in length and higher than 30X of coverage were selected for further analysis.

The Flye v2.9.4 (https://usegalaxy.org/) [15,17] was employed to assemble the long reads generated from ONT. The contigs generated from SPAdes were blasted against the contigs generated from Flye. The shared and unique contigs were used as a draft genome for further analysis. The short reads and long reads were mapped to corresponding contigs in the draft genome using minimap2 (https://usegalaxy.org/) [15,18]. The BAM files generated from minimap2 and short reads were subjected to polishing using Hypo v1.0.3 [19]. The short reads were mapped to sequences generated from Hypo, and the contigs were manually inspected using Geneious Prime, 2023 [20].

### Analysis of the assembled genome

#### Genome-based taxonomic analysis

The assembled genome of AG1 was subjected to ribosomal multilocus sequence typing (rMLST) and multilocus sequence typing (MLST) [21]. In addition, AG1 and *V. parahaemolyticus* (JS20200428004-2) were subjected to the type (strain) genome server (TYGS) (https://tygs.dsmz.de, accessed on 10 December 2024) for whole-genome-based taxonomic analysis with a digital DNA–DNA hybridization (dDDH) threshold of 70% [22]. The AG1 and JS20200428004-2 genomes were compared against the type strains from the TYGS database by using the MASH algorithm (a fast approximation of intergenomic relatedness) [23]. First, ten type strains with the smallest MASH distances were selected for each genome. Second, the 16S rDNA sequences were extracted from AG1 and JS20200428004-2 genomes using RNAmmer [23]. Then, they were searched using blast against 16S rDNA sequences from 22,144 type strains in the TYGS database [24]. This process allowed for the identification of the best 50 matching type strains for each genome. Subsequently, the precise distances used to determine the ten closest strains for each genome were calculated using the genome blast distance phylogeny (GBDP) approach under the algorithm ‘coverage’ and distance formula *d5* [25]. For phylogeny inference, the GBDP approach was used for all pairwise comparisons among genomes, and exact intergenomic distances were inferred using the algorithm ‘trimming’ and distance formula *d5*. A total of 100 distant replicates were computed for each comparison. The values of dDDH and their confidence intervals were calculated using the recommended settings of GGDC 4.0 [25,26].

#### Species and subspecies clustering

The dDDH was used to determine the species and subspecies clustering with cutoff values set at 70% for species clustering and 79% for subspecies clustering [27]. Moreover, the average nucleotide identity (ANI) between the studied strains and the closest strains was calculated using ANI (MUMmer algorithm) v.0.2.12 [28,29]. The ANI results were displayed using pheatmap v1.0.12 [30].

#### Phylogenetic analysis

The resulting intergenomic distances were employed for phylogenetic analysis to build a balanced minimum evolution tree with branch support via FASTME 2.1.6.1. In addition, the tree topology was optimized using post-processing of subtree pruning and regrafting [31]. Branch support was inferred based on 100 pseudo-bootstrap replicates. The trees were rooted at the midpoint and shown with the PhyD3 tool [32,33].

#### Antibiotic-resistant profiling

The acquired antibiotic-resistant genes from AG1 and JS20200428004-2 genomes were detected using ResFinder v.4.6.0 (http://genepi.food.dtu.dk/resfinder, accessed on 4 December 2024) [24,34] and ABRicate v1.0.0 using multiple databases, including NCBI, CARD, ARG-ANNOT, Resfinder, MEGARES, EcOH, PlasmidFinder, Ecoli_VF and VFDB, with an identical threshold set to 90% [35,43].

#### Mobile genetic element identification

The mobile genetic elements (MGEs) [i.e. insert sequence (IS), genomic island (GI) and transposase] from AG1 and JS20200428004-2 were identified using IS-Finder [blastn, E-value=0.0 (www-is.biotoul.fr)], IslandViewer 4 [44] and blastp.

#### Annotation and comparative genome analysis

The assembled genome was annotated using rapid annotation using subsystem technology (https://rast.nmpdr.org/, accessed on 20 November 2024) [45,47] and validated by prokaryotic genome annotation pipeline (pgap_2024-07-18.build7555) [48]. The protein domains were identified in the virulent plasmid of AG1 using the Interproscan package in Geneious Prime v2023 [20]. The genome sequences from AG1 and Js20200428004-2 were aligned using [20] blastn and illustrated using EasyFig [49]. In addition, the deduced amino acid sequences of virulent proteins (i.e. VHVP-1, -2 and -3) from AG1 and JS20200428004-2 were aligned using the MAFFT package (auto; BLOSUM62) via Geneious Prime v2023.

### Bioassay

Bioassay was conducted to demonstrate the pathogenicity of *V. parahaemolyticus* AG1. Briefly, 80 juvenile Specific Pathogen Free *P. vannamei* shrimp (avg. ~0.5 g) were put into four 4l jars (20 shrimp per jar) with aeration. Animals in two jars were challenged via immersion at 10^5^ c.f.u. ml^−1^ while the animals in the remaining two jars served as negative controls. The AG1 strain was grown in TSB containing 2% NaCl overnight at 30 °C and shaken at 150 r.p.m. The bacterial broth (OD_600_=3.5, ~2.8×10^9^ c.f.u. ml^−1^) was added to two jars at a final concentration of ~10^5^ c.f.u. ml^−1^. The experiment was terminated at 96 h post-inoculation (hpi). The mortality was recorded, and the survival analysis was executed using the Kaplan–Meier curve via the MedCalc program v22.2023. Moribund shrimp were fixed in Davidson’s AFA solution for histopathology analysis. These shrimp were dissected and embedded in paraffin blocks, which were subsequently sectioned and stained with Mayer-Bennett’s haematoxylin and eosin-phloxine (H and E). The slides were examined under the light microscope following Lightner’s grading scale to determine severity [50]. The DNA was extracted from moribund shrimp using the DNeasy Blood and Tissue kit (Qiagen, Germany). The extracted DNA was subjected to PCR for the detection of *vhvp-1* and *-2* using primers/probes described in a recently published paper [9] (Table S1).

## Results

### The TPD virulent gene detection

**Fig. 1.**
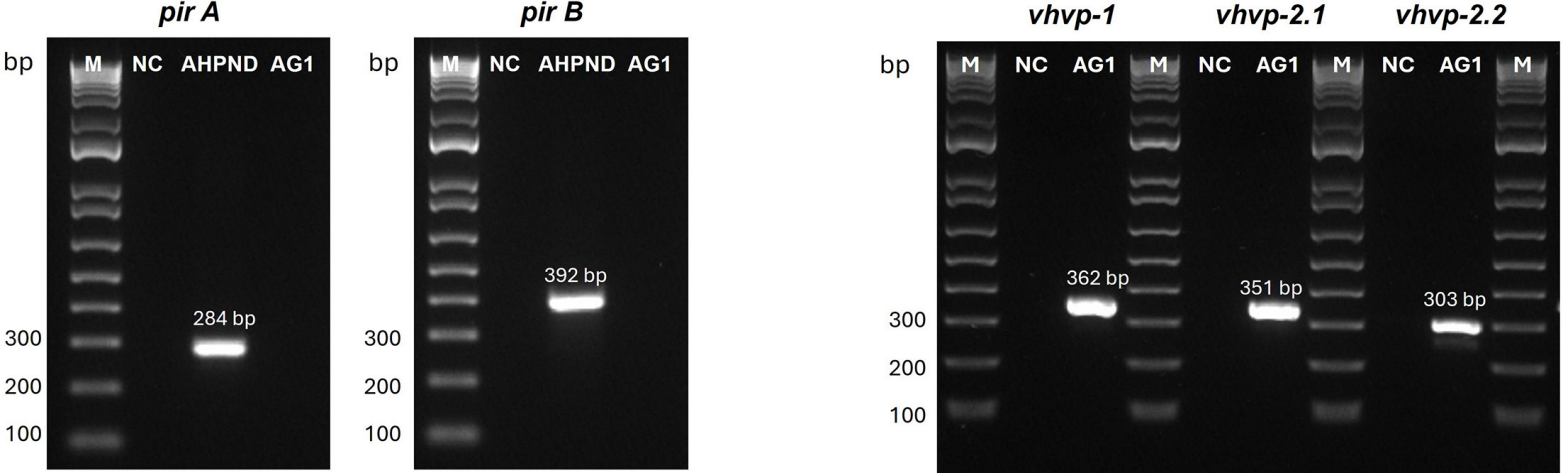
The detection of *pir*A, *pir*B, *vhvp-1*, *vhvp-2.1* and *vhvp-2.2* by conventional PCR assay in bacterial isolate AG1 isolated from *P. vannamei* post-larvae experiencing mortalities in a hatchery. M=Marker; NTC=non-template control.

The *vhvp-1* and *-2* genes were detected in *V. parahaemolyticus* isolate AG1 using conventional PCR, while the AHPND binary toxin-encoded genes, *pir*AB, could not be detected (Fig. 1). The results confirmed that the bacterial isolate, AG1, represents a non-AHPND-causing *V. parahaemolyticus*.

### Genome assembly

A total of 32,302,204 reads were generated from the Illumina platform. After adapter trimming and low-quality read removal, 32,177,487 reads were used for *de novo* assembling, generating 165 contigs (>500 bp each, N50=340,906 bp). Regarding long reads, 167,323 reads were generated from the Oxford Nanopore platform. The Flye-based assembly produced five sequences, ranging from 59,740 to 3,495,070 bp in length. Circular topology was detected in four out of five sequences. The sequences from 165 contigs generated from the short-read assembly were found in five sequences produced by the long-read assembly. Five circular sequences, including two chromosomes and three plasmids (average coverage ~759.16X), were obtained from Hypo-based polishing (Table 1).

### Genome-based taxonomic analysis

**Fig. 2.**
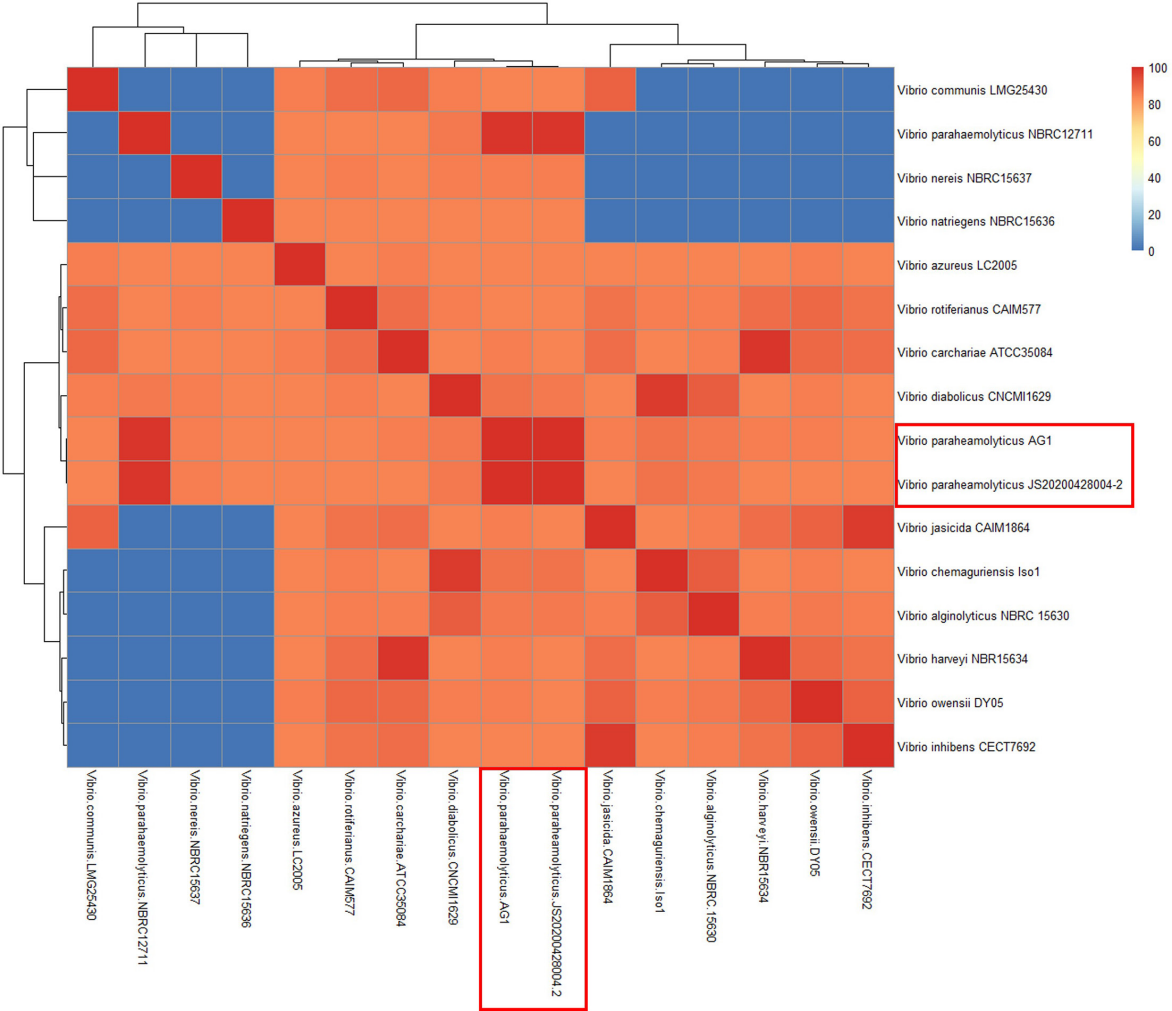
The ANI between *V. parahaemolyticus* AG1 and other related *Vibrio* species. The *V. parahaemolyticus* AG1 and JS20200428004-2 (*Vp*_TPD_) are in the red rectangle. The red colours indicate high identities, while the blue colours indicate low identities.

The multilocus typing sequence analysis per MLST and rMLST database revealed that the sequences of internal fragments of seven housekeeping genes (i.e. *dnaE*, *gyrB*, *recA*, *dtdS*, *pntA*, *pyrC* and *tnaA*) were 100% identical to MLST profile of * V. parahaemolyticus*. In addition, the dDDH analysis comparing genome sequences (i.e. AG1 and JS20200428004-2) and related strains in TYGS database showed that the dDDH values (*d4* formula) ranged from 21.9 to 99.3%. The dDDH value between AG1 and JS20200428004-2 was 99.3% with a confidence interval of (98.9–99.6) (Table 2). In addition, the pair comprising AG1 and JS20200428004-2 showed the highest value of ANI (Fig. 2).

**Table 2.** Pairwise comparisons of genomes of *V. parahaemolyticus* isolates AG1 and JS20200428004-2 genomes to type strain genomes from TYGS database

Studied strain	TYGS strain	dDDH (*d0*, in %)	CI (*d0*, in %)	dDDH (*d4*, in %)	CI (*d4*, in %)	dDDH (*d6*, in %)	CI (*d6*, in %)
‘*V. parahaemolyticus* AG1’	‘*V. parahaemolyticus*-JS20200428004-2’	96.4	[94.5–97.6]	99.3	[98.9–99.6]	98.1	[97.0–98.8]
‘*V. parahaemolyticus*-JS20200428004-2’	*V. parahaemolyticus* NBRC 12711	81.9	[78.0–85.2]	86.7	[84.1–88.9]	85.6	[82.5–88.3]
‘*V. parahaemolyticus* AG1’	*V. parahaemolyticus* NBRC 12711	86.7	[83.2–89.7]	86.5	[83.9–88.8]	89.5	[86.7–91.8]
‘*V. parahaemolyticus*-JS20200428004-2’	*Vibrio chemaguriensis* Iso1	60.3	[56.6–63.8]	27.9	[25.6–30.4]	50.2	[47.2–53.3]
‘*V. parahaemolyticus* AG1’	*V. chemaguriensis* Iso1	64.1	[60.3–67.7]	27.9	[25.6–30.4]	52.8	[49.7–55.9]
‘*V. parahaemolyticus* AG1’	*Vibrio diabolicus* CNCM I-1629	62.3	[58.6–65.9]	27.7	[25.3–30.2]	51.4	[48.3–54.5]
‘*V. parahaemolyticus*-JS20200428004-2’	*V. diabolicus* CNCM I-1629	58.5	[54.9–62.1]	27.7	[25.3–30.2]	48.9	[45.9–52.0]
‘*V. parahaemolyticus*-JS20200428004-2’	*Vibrio alginolyticus* NBRC 15630	53.2	[49.7–56.6]	26.6	[24.2–29.1]	44.7	[41.7–47.7]
‘*V. parahaemolyticus* AG1’	*V. alginolyticus* NBRC 15630	56.3	[52.8–59.8]	26.6	[24.2–29.1]	46.8	[43.8–49.8]
‘*V. parahaemolyticus*-JS20200428004-2’	*Vibrio owensii* DY05	34.9	[31.5–38.4]	24.4	[22.1–26.9]	31.2	[28.3–34.3]
‘*V. parahaemolyticus* AG1’	*V. owensii* DY05	35.7	[32.3–39.2]	24.3	[22.0–26.8]	31.8	[28.9–34.9]
‘*V. parahaemolyticus*-JS20200428004-2’	*Vibrio carchariae* ATCC 35084	37.7	[34.3–41.2]	24.2	[21.9–26.7]	33.1	[30.2–36.2]
‘*V. parahaemolyticus* AG1’	*V. carchariae* ATCC 35084	39.3	[35.9–42.7]	24.2	[21.9–26.7]	34.2	[31.3–37.3]
‘*V. parahaemolyticus*-JS20200428004-2’	*Vibrio jasicida* CAIM 1864	37.3	[33.9–40.8]	24.2	[21.9–26.6]	32.8	[29.9–35.9]
‘*V. parahaemolyticus* AG1’	*Vibrio communis* LMG 25430	39	[35.6–42.5]	24.1	[21.8–26.6]	34	[31.1–37.1]
‘*V. parahaemolyticus*-JS20200428004-2’	*V. communis* LMG 25430	37.4	[34.0–40.9]	24.1	[21.8–26.6]	32.9	[30.0–36.0]
‘*V. parahaemolyticus*-JS20200428004-2’	*Vibrio rotiferianus* CAIM 577	33.7	[30.3–37.2]	23.9	[21.6–26.3]	30.2	[27.3–33.3]
‘*V. parahaemolyticus* AG1’	*V. rotiferianus* CAIM 577	35	[31.6–38.5]	23.9	[21.5–26.3]	31.1	[28.2–34.2]
‘*V. parahaemolyticus* AG1’	*V. jasicida* CAIM 1864	38	[34.6–41.5]	23.9	[21.6–26.4]	33.3	[30.3–36.3]
‘*V. parahaemolyticus*-JS20200428004-2’	*Vibrio inhibens* CECT 7692	36	[32.6–39.5]	23.8	[21.5–26.3]	31.8	[28.9–34.9]
‘*V. parahaemolyticus*-JS20200428004-2’	*Vibrio harveyi* NBRC 15634	38.3	[34.9–41.8]	23.8	[21.5–26.3]	33.4	[30.5–36.5]
‘*V. parahaemolyticus* AG1’	*V. inhibens* CECT 7692	37.4	[34.1–40.9]	23.8	[21.5–26.3]	32.8	[29.9–35.9]
‘*V. parahaemolyticus* AG1’	*V. harveyi* NBRC 15634	40	[36.6–43.4]	23.8	[21.5–26.3]	34.5	[31.6–37.6]
‘*V. parahaemolyticus*-JS20200428004-2’	*Vibrio natriegens* NBRC 15636	33.4	[30.0–37.0]	23.7	[21.4–26.2]	30	[27.0–33.1]
‘*V. parahaemolyticus* AG1’	*V. natriegens* NBRC 15636	34.7	[31.3–38.2]	23.7	[21.4–26.2]	30.9	[27.9–34.0]
‘*V. parahaemolyticus* AG1’	*Vibrio hyugaensis* 090810 a	40.1	[36.7–43.6]	23.5	[21.2–26.0]	34.5	[31.6–37.6]
‘*V. parahaemolyticus*-JS20200428004-2’	*V. hyugaensis* 090810 a	38.3	[35.0–41.8]	23.5	[21.2–26.0]	33.3	[30.4–36.4]
‘*V. parahaemolyticus*-JS20200428004-2’	*Vibrio nereis* NBRC 15637	17.5	[14.5–21.0]	22.9	[20.6–25.3]	17.4	[14.8–20.4]
‘*V. parahaemolyticus* AG1’	*V. nereis* NBRC 15637	17.8	[14.7–21.3]	22.8	[20.5–25.3]	17.6	[15.0–20.6]
‘*V. parahaemolyticus*-JS20200428004-2’	*Vibrio azureus* NBRC 104587	17.8	[14.7–21.4]	21.9	[19.6–24.3]	17.6	[15.0–20.6]
‘*V. parahaemolyticus* AG1’	*V. azureus* NBRC 104587	18	[14.9–21.6]	21.9	[19.6–24.3]	17.8	[15.1–20.8]

The numbers (%) showed the dDDH values between studied genomes and type strain genome from TYGS database. The dDDH values were computed using three formula (*d0*, *d4* and *d6*).

CI, Confidence interval.

### Species and subspecies clustering

**Fig. 3.**
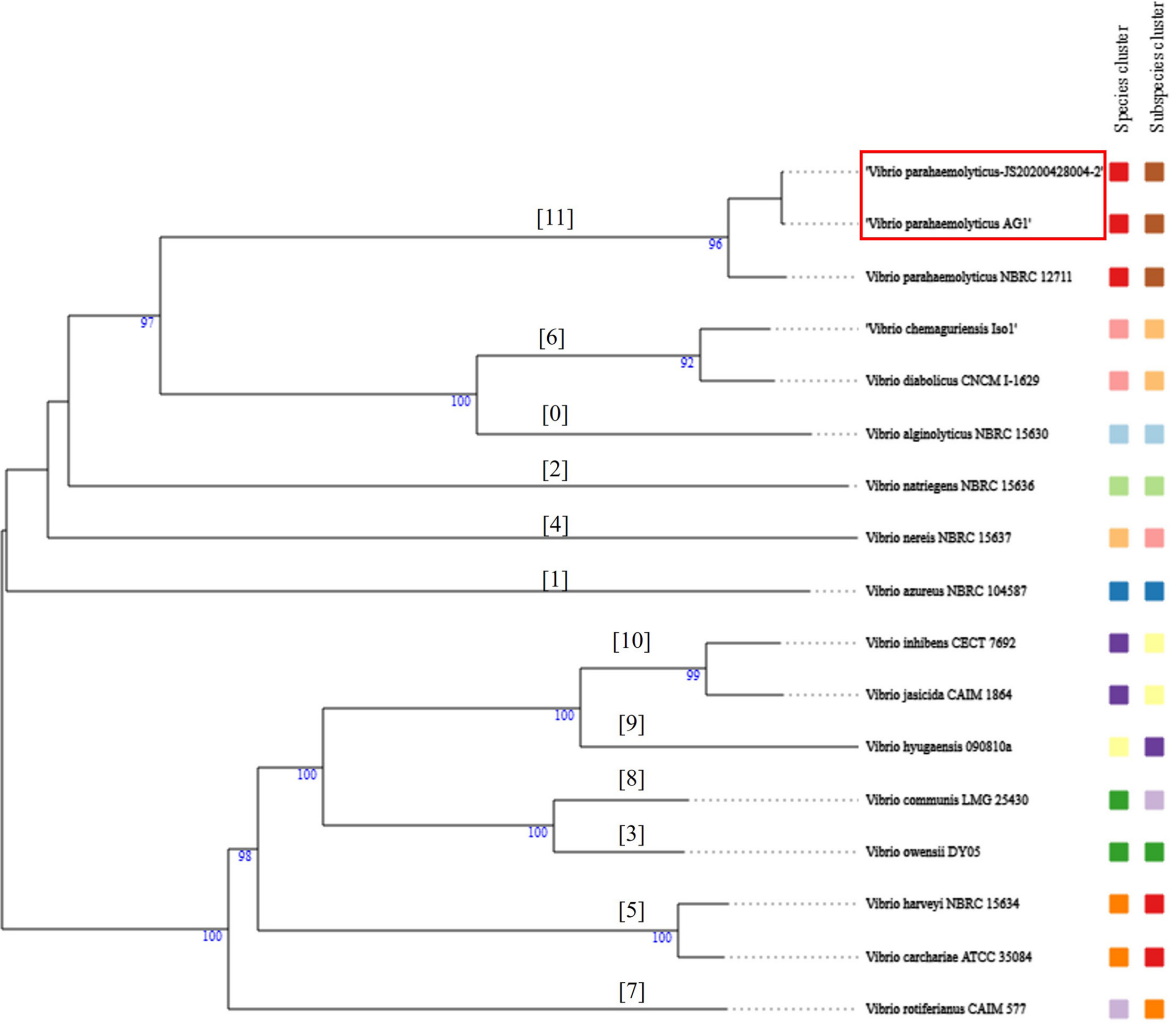
The phylogenetic tree using GBDP distances calculated from whole-genome sequence of *Vibrio* spp. The numbers indicate GBDP pseudo-bootstrap support values >60% from 100 replications. The *V. parahaemolyticus* AG1 and JS20200428004-2 (*Vp*_TPD_) are in the red rectangle. The same colours indicate the same species/subspecies clusters. The numbers in brackets indicate subspecies clusters.

Ten species clusters (1–11) were identified, and AG1, *Vp*_TPD_ and *V. parahaemolyticus* NBRC 12711 were in cluster #6. Subspecies clustering revealed that AG1, *Vp*_TPD_ and *V. parahaemolyticus* NBRC 12711 were also in the same subspecies cluster (#11) (Table S2 and Fig. 3).

### Phylogenetic analysis

The phylogenetic tree inferred from intergenomic distances showed that AG1, *Vp*_TPD_ and *V. parahaemolyticus* NBRC 12711 were in the same group, supported by a bootstrap value of 96 (Fig. 3).

### Antibiotic-resistant profile

The AG1 strain carried three antibiotic-resistant genes, including *qnrVC10*, *tet*(*35*) and *blaCARB-26*. The AG1 strain was predicted to be resistant to antibiotics belonging to several antibiotic classes, including quinolone, tetracycline and beta-lactam (Table 3). Interestingly, the number of antibiotic-resistant genes was higher in JS20200428004-2 than in AG1 (8 vs. 3). The antibiotic-resistant genes found in JS20200428004-2 included *tet*(*35*), *qnrVC10*, *blaCARB-26*, *sul2*, *aph(3′)-Ib*, *aph(6)-Id*, *tet*(*A*) and *floR*, making this strain potentially resistant to antibiotics (i.e. quinolone, beta-lactam, sulphonamide, streptomycin, tetracycline, chloramphenicol and florfenicol) (Table 3).

**Table 3.** Acquired antibiotic-resistant genes in AG1 and JS20200428004-2 The genome sequence of both strains was scanned for antibiotic-resistant genes using Resfinder and Abricate with identity threshold=90%.

Strain	Location	Position	Resistant gene	Identity%	Product resistance
*Vp*-AG1	Chromosome 1	448,560–449,216	*qnrVC10*	99.7	Quinolone
	Chromosome 1	1,264,786–1,266,387	*tet*(*35*)	98.5	Tetracycline
	Chromosome 2	470,452–471,303	*blaCARB-26*	99.18	Beta-lactam
*Vp*-JS20200428004-2	Chromosome 1	2,288,818–2,290,419	*tet*(*35*)	98.5	Tetracycline
	Chromosome 1	3,105,606–3,106,262	*qnrVC10*	99.7	Quinolone
	Chromosome 2	1,504,690–1,505,541	*blaCARB-26*	100	Beta-lactam
	Plasmid-1	76,920–77,735	*sul2*	100	Sulphonamide
	Plasmid-1	77,772–78,599	*aph(3′)-Ib*	100	Streptomycin
	Plasmid-1	78,599–79,435; 82,557–83,390	*aph(6)-Id*	100/99.64	Streptomycin
	Plasmid-1	80,090–81,289	*tet*(*A*)	100	Tetracycline
	Plasmid-1	84,346–85,560	*floR*	100	Chloramphenicol; florfenicol

### MGE identification

A total of four IS (i.e. ISVpa3, ISVvu6, ISVvu4 and ISVa19) were identified across the genome of AG1. Meanwhile, ISVpa3, ISVvsa3, ISVa19, ISVva6, ISVva3 and ISVvu4 were identified in the genome of JS20200428004-2 (Table S3). The GIs were also identified in the genomes of AG1 (30 GI) and JS20200428004-2 (33 GI) (Table S3).

### Comparative genomics

The AG1 strain genome contained 5,192 genes, including 4,948 genes encoding proteins, 173 genes for RNAs and 71 pseudogenes. The completed rRNAs (i.e. 5S, 16S and 23S) were successfully identified in the AG1 genome. The AG1 genome was deposited in GenBank under the accession numbers (CP176357–CP176361) (Table 1).

By comparison, the chromosome 1 from AG1 and JS20200428004-2 strains was similar. Those chromosomes shared two antibiotic-resistant genes [i.e. *qnrVC10* and *tet*(*35*)] (Table 3). Additionally, the number of IS identified on chromosome 1 was higher in JS20200428004-2 than AG1 (3 vs. 2). In contrast, the number of GIs was greater in chromosome 1 of AG1 compared to JS20200428004-2 (15 vs. 23) (Table S3). The chromosome 2 (CP176358) from AG1 showed reversed orientations with chromosome 2 (SRR23329176.2) in JS20200428004-2, even though they show similarity (Fig. S1). Moreover, the beta-lactam-resistant gene was found in chromosome 2 of both strains. Although only one IS was found on chromosome 2 from AG1 and JS20200428004-2, more GIs in chromosome 2 were identified in JS20200428004-2 than in AG1. In addition, plasmid-1 (SRR23329176.3) containing antibiotic-resistant genes was not found in AG1, while plasmid-2 (CP176360) was not detected in JS20200428004-2. The plasmid-3 (CP176361) from AG1 showed similarity with plasmid-3 in JS20200428004-2, but they are in reversed orientations (Fig. S1). Interestingly, one GI was found in plasmid-3 from AG1, but no GI was detected in plasmid-3 from JS20200428004-2 (Table S3). Additionally, the type IV secretion system (T4SS) related genes were found in all plasmids (CP176359–CP176361) in AG1, while only plasmid-2 and plasmid-3 (SRR23329176.4-SRR23329176.5) in JS20200428004-2 were found to contain the T4SS-associated genes.

**Fig. 4.**
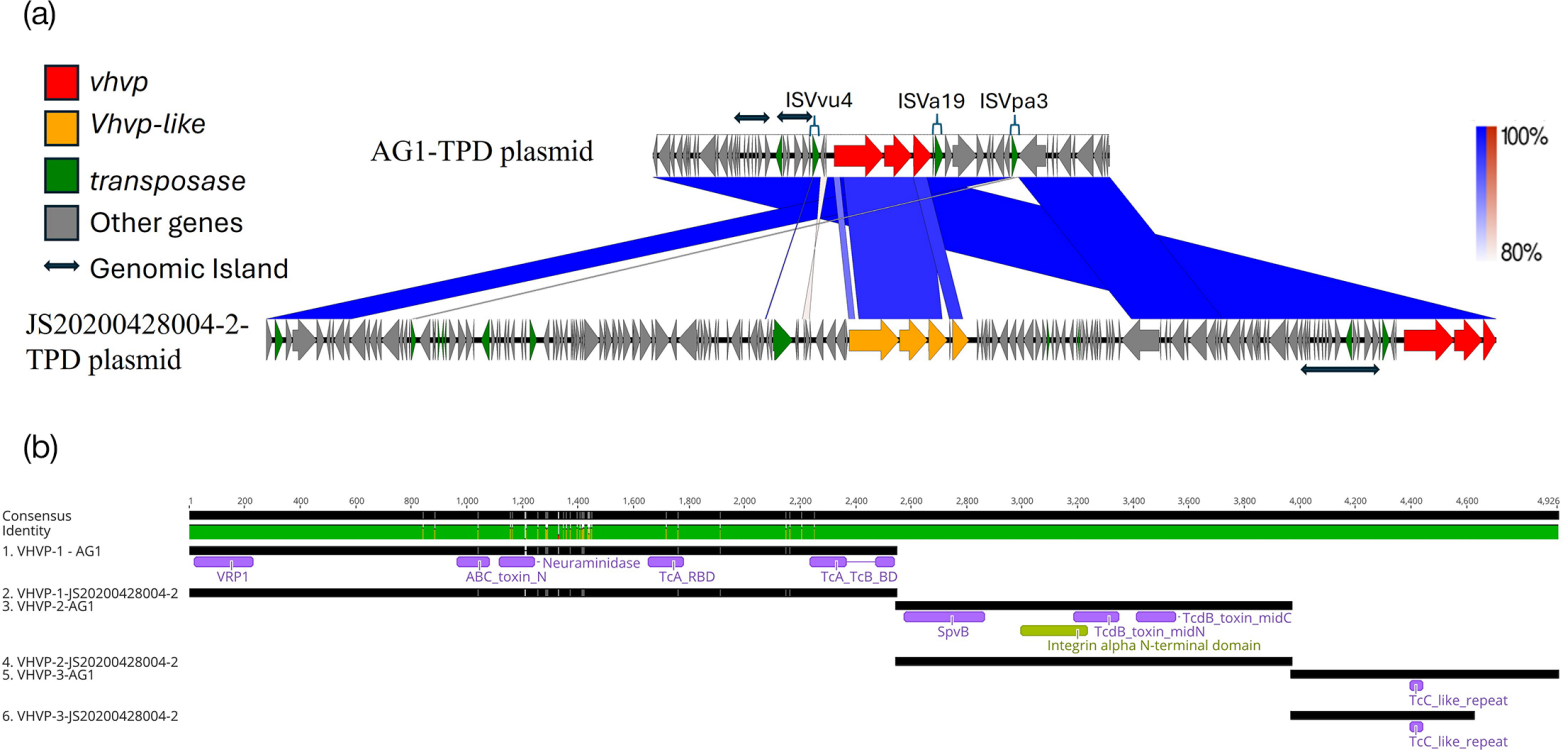
The genomic comparison of TPD plasmid between AG1 and JS20200428004-2. (**a**) The linear comparison of TPD plasmid between AG1 and JS20200428004-2. (**b**) The VHVP protein alignments between AG1 and JS20200428004-2. The green colours indicate identical amino acids. The yellow colours indicate mismatches.

The IS, transposases and GIs were identified in the VHVP-encoded plasmids in AG1 and JS20200428004-2. The *vhvp-1*, *-2* and *-3* were detected in ~69 kb plasmid of AG1 (Fig. 4a). Interestingly, the VHVP-encoding plasmid was significantly shorter in the AG1 strain than in the *Vp*-JS20200428004-2 strain (69,744 bp vs. 187,791 bp). Two cassettes of *vhvp* genes located at 83,809–98,809 and 173,714–187,791 were detected in SRR23329176.4 plasmid, while only one set of *vhvp* genes was detected in AG1 (Fig. 4a). Based on the VHVP-1 and 2 protein sequences provided in the previous publication [9], the *vhvp* genes located at 173,714–187,791 were likely to express VHVP proteins. In addition, the nucleotide sequences of *vhvp* genes from AG1 show high similarity (>90%) to *vhvp* genes in JS20200428004-2 (Fig. 4a).

The *vhvp-1*, *-2* and *-3* were 7,623, 4,266 and 2,886 bp in length, respectively. The VHVP-1, -2 and -3 comprised 2,540, 1,421 and 961 amino acids, respectively. The VHVP-1 contained domains of neuraminidase, insecticidal toxin, ABC toxin N-terminal, TcA receptor binding (TcA-RBD) and TcA and TcB binding (TcA-TcB-BD) (Fig. 4b). The VHVP-2 contained SpvB, integrin alpha N-terminal, TcdB toxin midN and TcdB toxin midC domains; meanwhile, the VHVP-3 contained rearrangement hotspot(RHS) repeat-associated core and TcC-like repeat domains (Fig. 4b). The deduced amino acid sequences from VHVP-2 and -3 from AG1 and *Vp*_TPD_ strains were 100% identical, whereas the VHVP-1 amino acid sequences between those strains were >98% identical. Interestingly, the VHVP-3 protein was longer in the AG1 strain than in JS20200428004-2 (961 aa vs. 657 aa) (Fig. 4b).

### Bioassay and histopathology

**Fig. 5.**
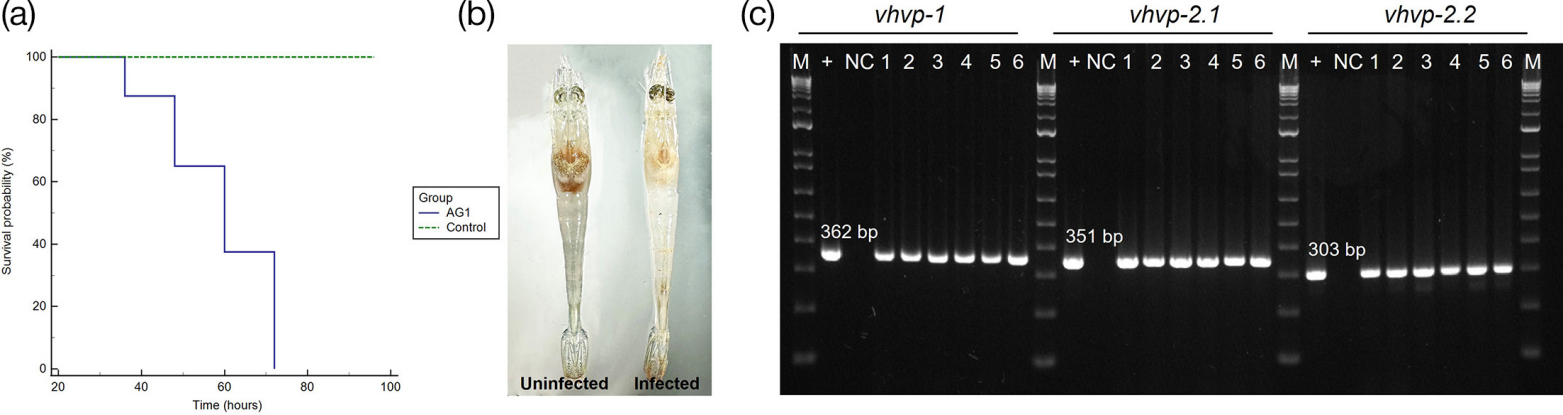
*P. vannamei* post-larvae challenged with *Vp*_TPD_*-*AG1. (**a**) The Kaplan–Meier graph shows survival probability. (**b**) The clinical signs of TPD-challenged *P. vannamei* post-larvae. (**c**) The detection of *vhvp* genes in *P. vannamei* post-larvae challenged with *Vp*_TPD_*-*AG1.

**Fig. 6.**
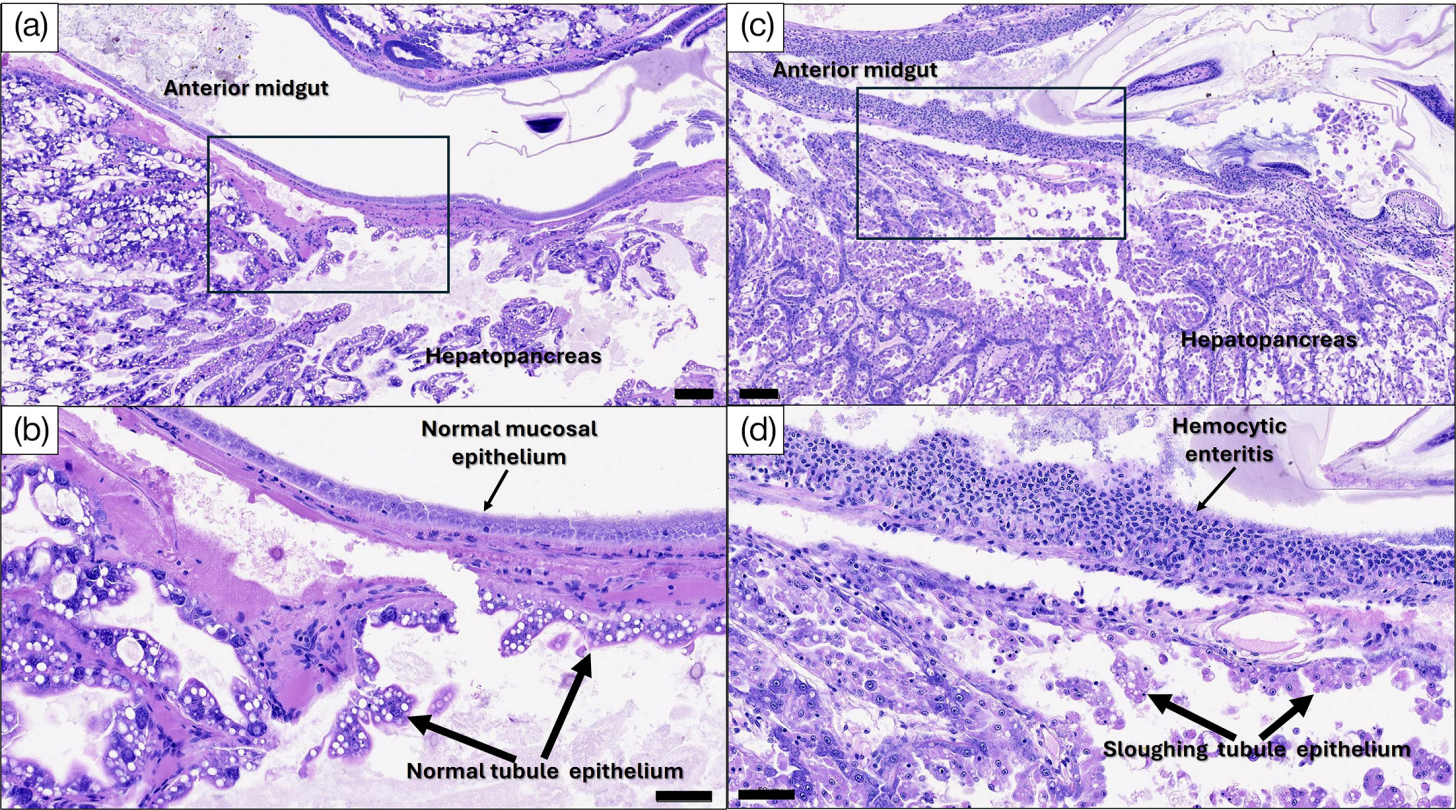
Histopathology of shrimp challenged with *Vp*_TPD_*-*AG1. (**a**) Normal appearance of hepatopancreas and anterior midgut in healthy shrimp from the negative control treatment. The area within the rectangle is shown at a higher magnification in (**b**). Scale bar=100 µm. (**b**) Notice the presence of intact mucosal epithelium in the midgut as well as normal tubule epithelial cells within the hepatopancreas. Scale bar=50 µm. (**c**) Panoramic view of a portion of hepatopancreas and anterior midgut from a *P. vannamei* post-larvae challenged with *Vp*_TPD_*-*AG1. The area within the rectangle is shown at a higher magnification in (**d**). Scale bar=100 µm. (**d**) Obvious loss of anterior midgut mucosal epithelium and replacement by a thick layer of haemocytes (HE). Severe sloughing of hepatopancreas tubule epithelial cells is also evident. Scale bar=50 µm. All pictures show staining with Mayer-Bennett’s haematoxylin/eosin-phloxine (H and E).

**Fig. 7.**
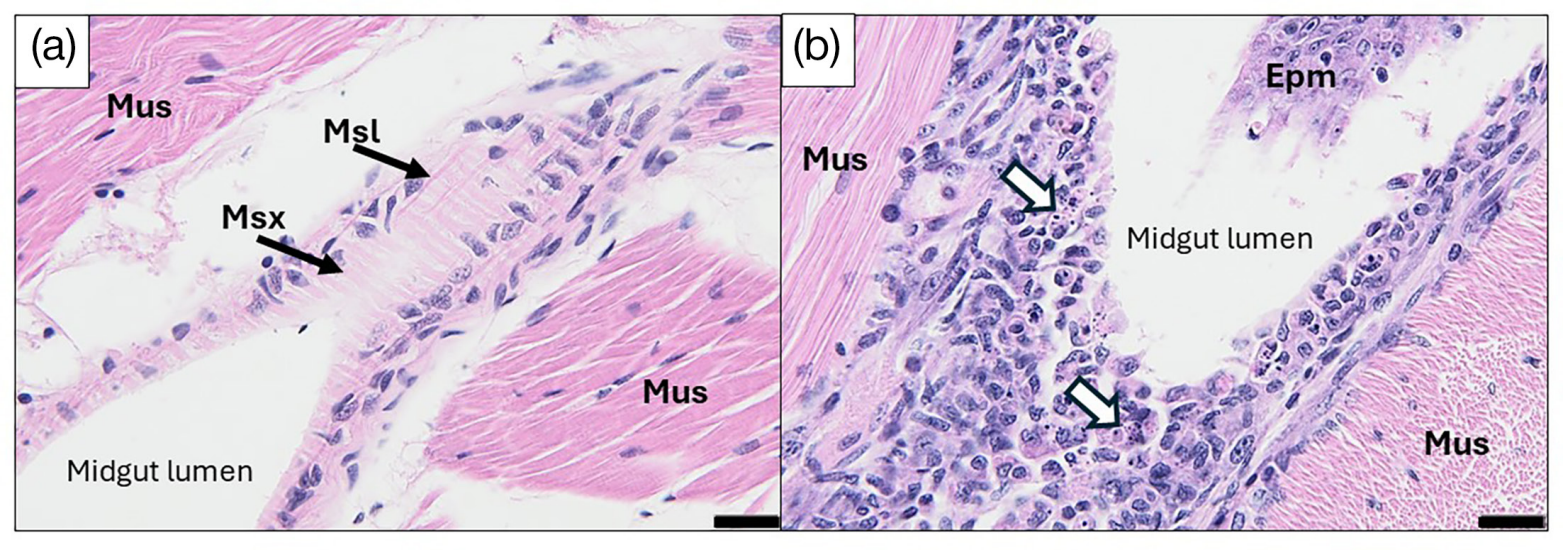
Histopathology of shrimp challenged with VpTPD-AG1. (**a**) Tangential section through the midgut of a healthy shrimp from the negative control treatment. The circular (Msx) and longitudinal (Msl) muscle layers are clearly visible and devoid of any lesions or abnormalities. The larger muscle bundles (Mus) are abdominal muscles. (**b**) Tangential section through the midgut of a shrimp challenged with VpTPD-AG1. The picture shows advanced necrosis of the circular and longitudinal muscle layers, with white arrows pointing to examples of pyknosis and karyorrhexis as well as haemocytic congestion. A portion of the mucosal epithelium (Epm) is visible in the upper right corner. Stain: Mayer-Bennett’s haematoxylin/eosin-phloxine (H and E). Scale bar=20 µm, both photographs.

Mortality reached 100% in *P. vannamei* post-larvae exposed to *Vp*_TPD_ via an immersion challenge method at 72 hpi (Fig. 5a). The *Vp*_TPD_-challenged shrimp displayed pale hepatopancreas and empty guts, whereas post-larvae that were not exposed to *Vp*_TPD_ displayed brown hepatopancreas and full guts (Fig. 5b). The *vhvp-1* and *vhvp-2* were detected in shrimp challenged with *Vp*_TPD_ (Fig. 5c). Histopathology of moribund animals showed ~43.6% of post-larvae exposed to *Vp*_TPD_ exhibited acute sloughing of hepatopancreatic tubule epithelial cells at severity levels that ranged from G1 to G4 [50]. In addition, ~87% of these shrimp presented with haemocytic enteritis (HE), which is characterized by loss of the midgut mucosal epithelium, eliciting a strong inflammatory reaction and the accumulation of a thick layer of haemocyte cells. HE severity levels ranged from G2 to G4 (Fig. 6c, d). None of these lesions were present in shrimp from the negative control treatment (Fig. 6a, b). Necrosis of the circular and longitudinal muscle layers that delimit the midgut was also observed in instances where tangential sectioning of the midgut exposed these muscle layers (Fig. 7a, b).

## Discussion

TPD, an emerging disease, was first reported in China in 2020. Recently, *V. parahaemolyticus* has been widely accepted as a causative agent of TPD, although an RNA virus, *Baishivirus*, a member of the family *Marnaviridae*, has also been reported as the aetiologic agent of TPD in *P. vannamei* shrimp [6,7]. The viral aetiology of TPD was reported based on metagenomic analysis without demonstrating River’s postulates. However, Koch’s postulate study revealed that a novel strain of *V. parahaemolyticus* carrying the virulent proteins causes the TPD in post-larvae (e.g. PL-5) of *P. vannamei* [9]. Recently, a few *V. parahaemolyticus* strains isolated from *P. vannamei* post-larvae experiencing mortalities in a hatchery in China were sequenced, and the genome sequence data are available in the GenBank database [9,12], but the genome of *Vp_TPD_* isolated from outside of China has not been fully characterized. In this study, *V. parahaemolyticus* was isolated from a TPD outbreak in a hatchery in Southeast Asia, and a single colony, AG1, was isolated in TCBS medium supplemented with 2% NaCl. The AG1 isolate showed the green colony in TCBS medium, suggesting AG1 represents a *V. parahaemolyticus* that does not ferment sucrose. In addition, *vhvp-1* and *-2* were detected in AG1, although *pir*A and *pir*B, the binary toxin genes of *V. parahaemolyticus* causing AHPND, could not be detected. This suggests *V. parahaemolyticus* isolate AG1 is likely the aetiologic agent of TPD.

The assembly of genome sequence data revealed that the genome of AG1 is ~5.6 Mb, which falls within the range of genome size reported for *Vibrio* species, i.e. 4.09–6.32 Mb [51]. In addition, the genome size of JS20200428004-2 reported from China is ~5.8 Mb [9], suggesting that AG1 was probably a *V. parahaemolyticus* causing TPD.

In the past, bacterial identification relied mainly on traditional biochemical tests, which are time-consuming methods and may not be able to differentiate the species accurately in some circumstances [52]. The development of high-throughput genome sequencing and informatic approaches builds the connections between genotypes and phenotypes, as it is found in the public databases such as MLST [21], rMLST [53] and TYGS [22]. Those databases contain valuable genomic information that is critical for accurately identifying an unknown bacterial species. In this study, the results from all three databases showed that AG1 represents a *V. parahaemolyticus* isolate. The results were also confirmed by ANI and dDDH analyses. It’s noteworthy that ANI and dDDH are both methods for complementing MLST in evaluating genomic relatedness [54]. A pairwise comparison of AG1 with other *Vibrio* species by ANI and dDDH analyses showed that the scores obtained for the pair of AG1 and *V. parahaemolyticus* were the highest. At the same time, the phylogenetic tree and species and subspecies clustering consistently showed that AG1 and *V. parahaemolyticus* NBRC 12711 strains were in the same group. Taken together, AG1 represents a *V. parahaemolyticus* isolate.

A bacterium can resist one or many antibiotics through mutations or acquiring antibiotic-resistant genes from other bacteria through vertical and horizontal transmission [55]. It is now well known that widespread and overuse of antibiotics leads to the emergence of antibiotic-resistant bacteria. In aquaculture, the presence of antimicrobial-resistant genes reflects the utilization of antibiotics in farming activities [56]. Analysis of the genome sequence data of the AG1 isolate revealed the presence of antibiotic-resistant genes against quinolone, tetracycline and beta-lactam. However, quinolone, beta-lactam and tetracycline-resistant genes [i.e. *qnrVC10*, *blaCARB-26* and *tet*(*35*)] were found only in chromosomes of AG1 and JS20200428004-2, suggesting that these strains might inherit those antibiotic-resistant genes from a common ancestor through vertical transmission.

MGEs, including plasmids, insertion sequences, transposons and GIs, facilitate the transmission of virulent factors and antibiotic-resistant genes among bacteria, thereby promoting genetic diversity and adaptability [57]. The plasmid-1 (SRR23329176.3) found only in the JS20200428004-2 strain contained several antibiotic-resistant genes [i.e. *sul2*, *aph(3′)-lb*, *aph(6)-ld*, *tet*(*A*) and *floR*], IS, transposase and GIs, suggesting that this plasmid might be obtained through horizontal transmission from the environment where several multidrug-resistant bacteria exist. Meanwhile, AG1 did not possess any antibiotic-resistant gene harboured plasmid, suggesting that the horizontal transmission of antibiotic-resistant genes might not have happened. In addition, the multidrug-resistant genes found in plasmid-1 (SRR2332976.3) may cause the higher tolerance of JS20200428004-2 to antibiotics compared to AG1. Therefore, this allows it to disseminate virulent factors to other bacteria. In other words, JS20200428004-2 may have higher virulence than AG1.

Although the ISs and GIs were detected in both the AG1 and JS20200428004-2 strains, the latter had more IS types and GIs than the former, suggesting that the environment from which JS20200428004-2 was isolated was more diverse. The plasmid-2 and -3 of AG1 were found to contain GI regions. Still, no IS were detected in plasmid-2 and -3, suggesting that those GIs were probably a non-autonomous GI that cannot move independently [58]. Although plasmid-3 from AG1 and JS20200428004-2 showed high similarity, only plasmid-3 from AG1 contained the GI. The possible reason could be the 12 bp gap in plasmid-3 between AG1 and JS20200428004-2, which might alter the protein sequence and result in no GI being detected in plasmid-3 of JS20200428004-2 strain. Interestingly, the transfer genes involved in T4SS were identified in plasmids from both strains. These results are in agreement with the study conducted by Zhang *et al.* [59], showing that T4SS has been identified in all 16 *Vp*_TPD_ strains. In addition, conjugative transfer genes were detected in all plasmids from the AG1 strain, suggesting these plasmids were responsible for the transmission of virulent factors and antibiotic-resistant genes to other bacteria. The AG1 isolate contains a VHVP-encoding plasmid, which is ~one-third of the length of the VHVP-encoding plasmid found in *Vp*_TPD_ reported from China. A possible explanation is the presence of a GI in the VHVP-encoding plasmid (TPD plasmid) of JS20200428004-2, indicating that the acquisition of foreign DNA has occurred [9,60]. Additionally, T4SS transfer genes were found in *vhvp* plasmids in both strains, which also facilitate the dissemination of vhvp genes. Indeed, *vhvp* genes were detected in different species of *Vibrio*, causing TPD in shrimp in China [9,61]. Interestingly, the number of cassettes of vhvp genes per TPD plasmid was doubled compared to other *Vp*_TPD_ strains [10,12], including AG1 strains.

The multi-subunit toxin complex (Tc) was first identified in the entomopathogenic bacterium, *Photorhabdus luminescens*. Subsequently, the Tc complex has been used to develop a biopesticide replacing Cry toxins released from *Bacillus thuringiensis* (*Bt*) in protecting plants from insects [62]. The domains of the Tc toxin complex were found in VHVP-1, -2 and -3 genes in both AG1 and JS20200428004-2. The Tc toxin complex, comprised of three components (i.e. TcA, TcB and TcC) that are known to have insecticidal activities [14]. In addition, all three components are needed to exert toxicity [63,64]. The TcA is responsible for injecting the actual toxin component into the host cells. In addition, TcA has a channel surrounded by a shell containing receptor-binding domains (TcA_RBD) responsible for binding to the receptors in the host cells [65]. Moreover, TcA is linked to the complex (made of TcB-TcC, called ‘cocoon’) via the TcA_TcB binding domain (TcA_TcB_BD) [65]. Those binding domains and VRP1 (a conserved domain in TcA) were also found in VHVP-1, suggesting that VHVP-1 might be responsible for receptor binding. The VHVP-2 in AG1 and JS20200428004-2 have identical amino acid sequences, suggesting they share similar functions. In addition, the conserved domains of TcB, including SpvB (located in the N-terminal) and TcdB_toxin_midN/C (located in the middle), were found in VHVP-2 in both AG1 and JS20200428004-2 isolated from Southeast Asia and China, respectively. The N-terminal 361 aa of TcB, in which SpvB is not critical for the toxicity, but they affect the expression level of TcB and TcC [66]. In *Salmonella*, however, SpvB shows ADP-ribosylate actin that prevents the conversion of G-actin to F-actin via polymerization, resulting in morphologic changes and loss of actin filaments in Madin-Darby Canine Kidney(MDCK) cells [67]. A cocoon formed by TcB and TcC contains the toxin component, the C-terminal hypervariable region (HVR) of TcC, which is cleaved autoproteolytically [65]. The binding of the cocoon to TcA via a six-bladed *β*-propeller from TcB triggers the cocoon’s opening and the toxin’s translocation into the channel [68], which is then injected into the cell [69]. Since different HVRs have distinct mechanisms of toxicity, the diversity of HVRs contributes to the virulence of the Tc toxin [69]. Interestingly, VHVP-3 from both strains also contained the RHS-associated core domain, which is conserved in the TcC component. However, the VHVP-3 in AG1 showed the HVR was ~30 kDa, which is in the range of HVR of TcC [22]. Meanwhile, VHVP-3 in JS20200428004-2 does not contain HVR, suggesting that the VHVP complex proteins from AG1 and JS2020428004-2 might have different targets and functions, resulting in bioassay outcomes.

Taken together, the VHVP complex protein may have a mechanism of toxicity similar to that of Tc toxin complex. We hypothesized that all three components, VHVP-1, -2 and -3, are needed to cause massive mortality in shrimp. The study done by Jia *et al.* in 2024 [11] showed that *Vibrio* species containing three components of VHVP cause 100% mortality; meanwhile, the bacteria with VHVP-1 and -2 or VHVP-2 alone cause 10–15% mortality, and the bacteria with VHVP-2 and -3 cause 100% mortality, suggesting VHVP-2 might not be the critical virulence factor contributing to the mortality in shrimp.

The experimental bioassay results showed that AG1 caused more mortalities in older shrimp than *Vp*_TPD_ did (juvenile vs. post-larvae 5). The *vhvp* genes were detected by PCR in AG1-challenged shrimp, suggesting they got infected by AG1. Histopathology analysis of moribund shrimp challenged with AG1 displayed lesions in the hepatopancreas, which are similar to the lesions caused by JS20200428004-2 [9]. This finding once again confirmed that AG1 bacterium is the causal agent of TPD in *P. vannamei*. In addition, HE was observed in the midgut of the majority of challenged shrimp, suggesting both the hepatopancreas and midgut are the target organs. In the case of the midgut, the lesions are not limited to the mucosal epithelium but also extend into underlying circular and longitudinal muscle layers, where we observed necrosis and inflammation. These observations agree with those recently reported by Srisala *et al.* [70]. This is the first time that HE has been linked to TPD. Recent publications on TPD do not mention the presence of HE nor its relation to this disease.

In summary, we isolated a bacterium from the post-larvae of *P. vannamei* shrimp experiencing mortalities in the hatchery, and the genome of a pure culture bacterial isolate, AG1, was sequenced. The genome of AG1 is ~5.5 Mb, consisting of two chromosomes and three plasmids, including plasmids that carry genes that encode virulence factors VHVP-1, VHVP-2 and VHVP-3 for causing TPD. Interestingly, genomic characterization showed that the VHVP proteins from AG1 are probably a Tc-like toxin complex like the *Salmonella* toxin. The genome-based taxonomy revealed that AG1 represents *V. parahaemolyticus*. Experimental bioassay using Specific Pathogen-Free(SPF) *P. vannamei* post-larvae and an immersion challenge method showed AG1 isolate can cause mortality up to 100%. The presence of HE in experimentally challenged shrimp post-larvae is a new histopathological finding in the study of TPD. Combining histopathology and PCR-based assay will be useful in the diagnosis of TPD in shrimp.

## Supplementary material

10.1099/mgen.0.001570 Fig. S1.

10.1099/mgen.0.001570 Uncited Supplementary Material 1.

10.1099/mgen.0.001570 Uncited Table S1.

## Abbreviations

AHPND: Acute hepatopancreatic necrosis disease
ANI: average nucleotide identity
dDDH: digital DNA–DNA hybridization
GBDP: genome blast distance phylogeny
GI: genomic island
HE: haemocytic enteritis
hpi: Hours post-inoculation
HVR: hypervariable region
IS: insert sequence
MGEs: mobile genetic elements
MLST: multilocus sequence typing
ONT: Oxford Nanopore Technologies
RHS: rearrangement hotspot
rMLST: ribosomal multilocus sequence typing
TCBS: Thiosulfate Citrate Bile Salts Sucrose
TPD: translucent post-larvae disease
TSB: Tryptic soy broth
T4SS: type IV secretion system
TYGS: type (strain) genome server
VHVP: Vibrio High Virulent Protein
