# Supplementary material for: Genomic characterization of Vibrio parahaemolyticus strain (AG1) causing translucent post-larvae disease in Penaeus vannamei
Source: Microb Genom. 2025 Dec 1;11(12):001570. doi: 10.1099/mgen.0.001570 (PMC12668620; doi:10.1099/mgen.0.001570)
Supplement: Fig. S1. [file mgen-11-01570-s001.pdf]

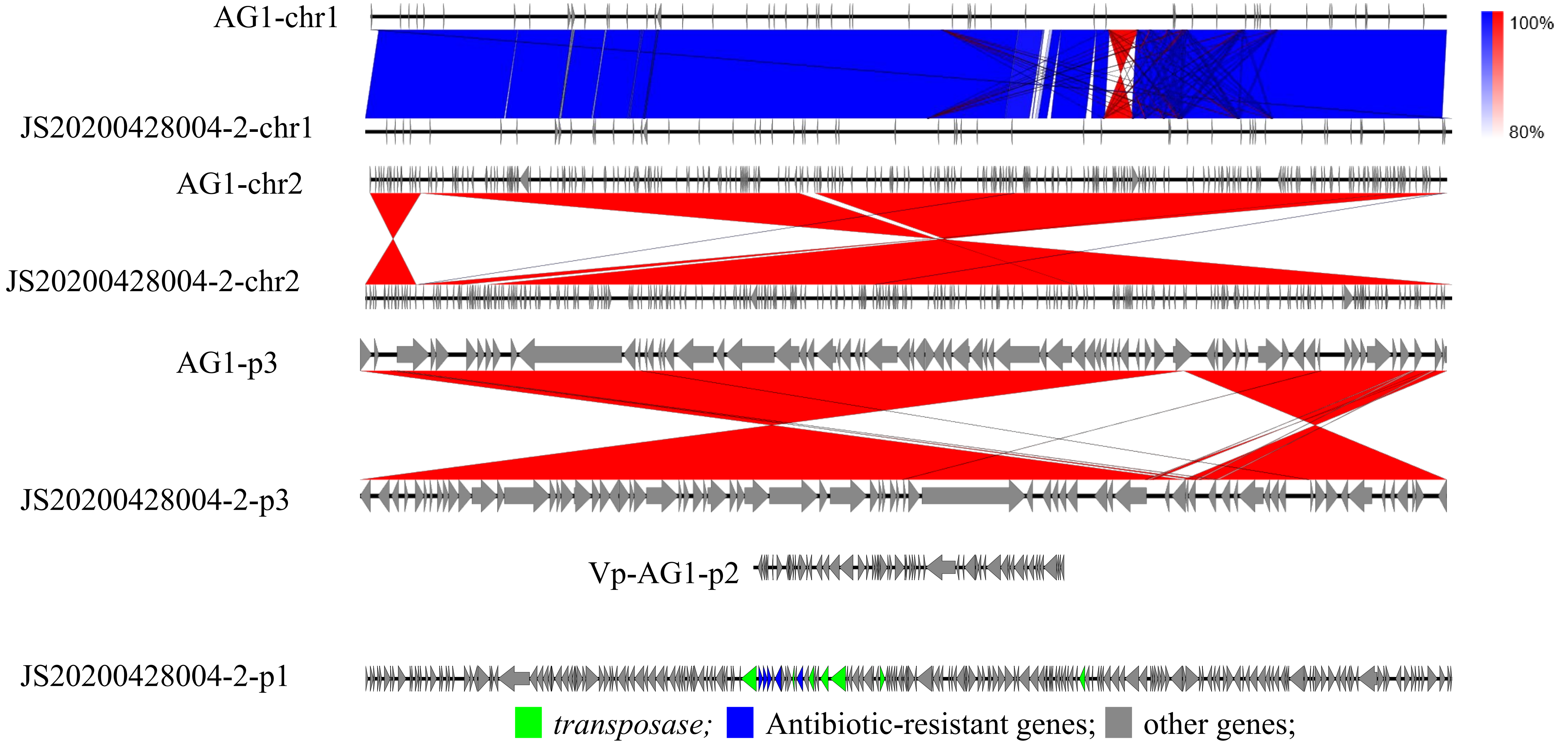

Fig S1. Linear comparison between AG1 and JS20200428004-2 genomes (except TPD plasmids). The blue color indicates that the conserved regions are in the same direction; The red color indicates that the conserved regions are inverted
